# Inferring Destinations and Activity Types of Older Adults From GPS Data: Algorithm Development and Validation

**DOI:** 10.2196/18008

**Published:** 2020-07-28

**Authors:** Sayeh Bayat, Gary Naglie, Mark J Rapoport, Elaine Stasiulis, Belkacem Chikhaoui, Alex Mihailidis

**Affiliations:** 1 Institute of Biomaterials and Biomedical Engineering University of Toronto Toronto, ON Canada; 2 KITE Research Institute Toronto Rehabilitation Institute University Health Network Toronto, ON Canada; 3 Department of Medicine Baycrest Health Sciences Toronto, ON Canada; 4 Department of Medicine University of Toronto Toronto, ON Canada; 5 Rotman Research Institute Baycrest Health Sciences Toronto, ON Canada; 6 Institute of Health Policy, Management and Evaluation University of Toronto Toronto, ON Canada; 7 Department of Psychiatry Sunnybrook Health Sciences Centre Toronto, ON Canada; 8 Department of Psychiatry University of Toronto Toronto, ON Canada; 9 Institute of Medical Science University of Toronto Toronto, ON Canada; 10 Laboratoire en Informatique Cognitive et Environnements de Formation Research Institute Department of Science and Technology TELUQ University Montreal, QC Canada; 11 Department of Occupational Therapy and Occupational Science University of Toronto Toronto, ON Canada

**Keywords:** outdoor mobility, older adults, GPS, life space, activity types, machine learning

## Abstract

**Background:**

Outdoor mobility is an important aspect of older adults’ functional status. GPS has been used to create indicators reflecting the spatiotemporal dimensions of outdoor mobility for applications in health and aging. However, outdoor mobility is a multidimensional construct. There is, as of yet, no classification algorithm that groups and characterizes older adults’ outdoor mobility based on its semantic aspects (ie, mobility intentions and motivations) by integrating geographic and domain knowledge.

**Objective:**

This study assesses the feasibility of using GPS to determine semantic dimensions of older adults’ outdoor mobility, including destinations and activity types.

**Methods:**

A total of 5 healthy individuals, aged 65 years or older, carried a GPS device when traveling outside their homes for 4 weeks. The participants were also given a travel diary to record details of all excursions from their homes, including date, time, and destination information. We first designed and implemented an algorithm to extract destinations and infer activity types (eg, food, shopping, and sport) from the GPS data. We then evaluated the performance of the GPS-derived destination and activity information against the traditional diary method.

**Results:**

Our results detected the stop locations of older adults from their GPS data with an F1 score of 87%. On average, the extracted home locations were within a 40.18-meter (SD 1.18) distance of the actual home locations. For the activity-inference algorithm, our results reached an F1 score of 86% for all participants, suggesting a reasonable accuracy against the travel diary recordings. Our results also suggest that the activity inference’s accuracy measure differed by neighborhood characteristics (ie, Walk Score).

**Conclusions:**

We conclude that GPS technology is accurate for determining semantic dimensions of outdoor mobility. However, further improvements may be needed to develop a robust application of this system that can be adopted in clinical practice.

## Introduction

With the growing population of older adults, the notion of *aging in place*, defined as “the ability to live in one's own home and community safely, independently, and comfortably,” is of increasing importance [1]. Aging in place is desirable from both individual quality-of-life and public health perspectives. Many older adults prefer to age in place because it enables them to preserve their independence, autonomy, and social connection [2]. Furthermore, aging in place is favored by policy makers and health providers because keeping people in their homes for as long as possible can reduce institutional care costs [3]. Although most studies on aging in place focus on the home environment, there is rising recognition about the importance of neighborhoods and communities in older adults’ ability to age in place [3-5]. Thus, many clinicians and researchers in the field of environmental gerontology are in search of a better understanding of older adults’ travel patterns in the outdoor environment, a concept that will be referred to as *outdoor mobility* throughout the rest of this paper.

A commonly used model that focuses on older adults’ outdoor mobility is *life space*, which is defined as the geographical area through which an individual moves [6]. To measure life space, the spatial environment is divided into several concentric zones (eg, bedroom, home, neighborhood, town, and out of town) and frequency of travel into each zone is recorded. Life space is traditionally measured through self-reported daily diaries or recall-based questionnaires [6,7]. The traditional life-space measures have many limitations. Travel diaries, for example, place the burden of data collection on study participants, which results in participants not recording trips that are too short or are made at inconvenient times. On the other hand, questionnaires require study participants to recall their movements during the month prior to the assessment date, which is particularly challenging for the cognitively impaired population. To overcome these limitations, more recent studies have used GPS data to create mobility indicators to assess life space, including maximum distance travelled, number of trips away from home, and area [8-11]. While most life-space studies rely only on a few indicators that represent spatial and temporal aspects of an individual’s mobility [12-14], it has become clearer that mobility is a multidimensional construct [12,15,16]. Thus, in order to understand the full range of properties that emerge from mobility patterns, the construct must encapsulate the spatial, temporal, and semantic dimensions. In the current life-space mobility measures, semantic dimensions that would enable an exploration of the reasons behind the patterns that emerge in space and time are not included.

According to the *travel demand model*, travel is a derived demand, meaning that individuals travel to specific destinations in their environment in order to participate in certain activities [17]. Older adults’ specific types of destinations and activities are indicative of their financial, psychosocial, physical, and cognitive status [18]. For example, participation in cognitively demanding activities, such as going to a bank, or physically demanding activities, such as playing tennis, can relay information about the cognitive or physical health of the individual, respectively. Furthermore, an understanding of relevant destinations and activities of older adults is required to design environments that support and target effective interventions that promote older adults’ outdoor mobility [18]. Therefore, examining the destinations that are relevant to older adults, as well as activities that older adults conduct at those destinations, is critical to the comprehensive understanding of their mobility behaviors.

However, the raw positioning data collected by GPS devices do not provide any additional contextual information, such as the places that people visit or the activities they perform. In travel behavior research, this information is traditionally collected through questionnaires where users are asked to annotate their trajectories. In the last few years, studies have increasingly aimed at automatically annotating raw GPS data with activities performed by users. The most popular method for automatically annotating GPS data in the existing travel behavior research is the rule-based method [19-23]. The available studies utilizing a rule-based method matched the GPS data with a series of predefined heuristic rules to determine the appropriate activity type. For example, Bohte and Maat [22] used a distance measure as a rule to determine the location that is being visited and the type of activity. They first obtained the location where a trip ends. If this location was within a radius of 50 m from a known location, it is assumed that this is the location that the user visited; otherwise, it was flagged as unknown. Furthermore, a number of studies used a probabilistic method to infer activity type [24-26]. These studies calculated the probability of participating in each of the potential activities according to a predefined measure and then selected the most probable activity type. For example, Furletti et al [27] used car trajectories to infer the *point of interest* the user has visited. They then inferred the activity performed using the category of the point of interest and a probability measure based on the gravity law. These existing studies, however, have only focused on identifying destinations and inferring activity types at the aggregated level and have failed to examine mobility at individual levels.

Furthermore, there has been little work done to date on characterizing older adults’ outdoor mobility according to its semantic aspects, such as destinations and activities. One approach entailed collecting activity-type information using self-report questionnaires [28]. This approach depends on the motivation and ability of the person to answer the questions accurately. An alternative approach has been to use ecological momentary assessments (EMAs) to classify types of activities. For example, in one study, activities of older adults within both residential and nonresidential environments were analyzed using EMAs [4]. That study was conducted during the course of 4 days, where EMA questions appeared as text on participants’ iPhone screens for them to answer. The data in this study included a sample of participants’ locations and activities within particular time windows and, therefore, was not comprehensive. In addition, EMA procedures can be burdensome and time consuming if the frequency of prompts increases [29]. Finally, both traditional questionnaires and EMAs are usually completed only a few times with each person and cannot effectively provide a comprehensive and objective assessment over time [30]. To date, however, no study has used an automated GPS-based approach to characterize older adults’ outdoor mobility according to its contextual information, such as destinations and activities. This study aims to develop and validate an inclusive and automated GPS-based outdoor mobility model for older adults that (1) extracts ones’ destinations and (2) infers activity types conducted at the most relevant destinations.

## Methods

### Data Collection

A total of 5 individuals [31], 65 years of age or older, were recruited from a registry of potential research participants that is maintained at Baycrest Health Sciences in Toronto, Ontario. Exclusion criteria included cognitive impairment as determined by a Montreal Cognitive Assessment score of less than 26 [32], significant functional limitations in activities of daily living (ADL) or instrumental activities of daily living (IADL) [33], and residing in assisted-living or senior housing. Informed consent was obtained from all participants. Ethics approval was obtained from the Baycrest Hospital Ethics Committee and the University Health Network Ethics Committee.

Participants were introduced to the SafeTracks Prime Mobile GPS device (SafeTracks GPS Canada) and instructed on how to use and charge it. The Prime Mobile device is small (3 cm 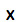 5 cm 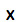 2 cm) and light (less than 100 g). It automatically starts tracking when turned on, and its battery life lasts for about 8 hours of continuous tracking. Participants were asked to place these devices in their pockets, purses, or bags, or to wear them around their neck as a pendant. They were also provided with a small booklet and were instructed to record details describing all excursions from their home, including time and destination information. All participants completed 4 weeks of GPS data collection and travel diary recording. The 4-week study period was selected based on the time period used in the traditional life-space assessments [6,7].

The GPS device recorded location (ie, longitude and latitude coordinates), speed, and heading direction with corresponding time and date stamps. While in the *motion state*, the device provided sensor readings at a frequency of one reading per minute, and while in the *nonmotion state*, the device transitioned into standby mode until motion was detected. Textbox 1 introduces the definitions and notations that are used throughout this paper.

**Terminology used in this paper.**

Definition 1: *GPS Record (P)* is the spatiotemporal location of the user in the form of (*Lat, Lon, t,* and *v*), where *Lat* and *Lon* are the latitude and longitude coordinates, *t* is the date-time stamp, and *v* is the instantaneous speed.

Definition 2: *Trajectory (T)* is a set of GPS records that are ordered based on their date-time attributes *t*,

*T* = {*P*_0_, *P*_1_, ... , *P_n_*}, where *t*_0_ < *t*_1_ < ... < *t_n_*

Definition 3: *Stop (S)* is a location at which the individual stays for more than a predefined time,

*S* = (*Lat*, *Lon*, *Δt)*

where *Δt* is the time the individual spent at the location, and the *Lat* and *Lon* coordinates are the centroid of all GPS points collected at the stop.

Since the stop measurements in the same location can vary, a stop cluster is defined.

Definition 4: *Stop Cluster* is a set of stops that belong to the same location.

### Stop Detection

#### Algorithm

**Figure 1:**
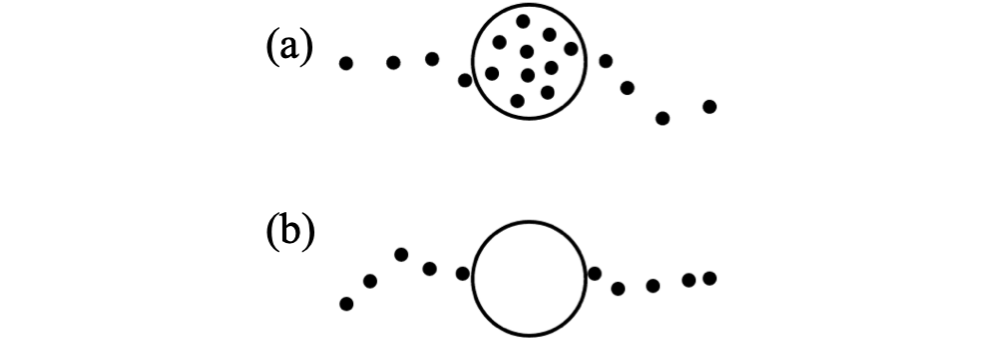
Stop types: (a) full-signal stop and (b) no-signal stop.

Two types of stops were considered when constructing a system to find the geographical locations of relevant destinations (ie, stops): (1) *full signal* and (2) *no signal* (see Figure 1). A *full-signal* stop was a stop location that contains a set of consecutive GPS records with no signal loss. The latitude and longitude of a full-signal stop was the centroid of all the GPS records in the stop. On the other hand, a *no-signal* stop was detected in two cases: (1) at areas with obstructed GPS signal and (2) when no motion is detected and the GPS device transitions into power-saving mode. This stop type consists of two GPS points: one immediately before and one immediately after the signal loss. The distance between these two adjacent points must be smaller than 150 meters. Otherwise, the signal loss can be due to underground transportation. The latitude and longitude of a *no-signal* stop was the centroid of the two GPS records in the stop. The velocity threshold was neglected because the GPS records in a no-signal stop may belong to the trip prior to the stop and poststop.

The stop-detection method used three threshold values. First, the time threshold (*δ_t_*) was set to 3 minutes to disregard short stops, such as stops at traffic lights, and to only detect the more meaningful destinations. Second, the distance threshold (*δ_d_*) was selected to be 150 meters based on the average block size in the Greater Toronto Area. Third, the speed threshold (*δ_v_*) was set to 2 m/s based on the SafeTracks GPS device speed–recording accuracy and the average walking speed for community-dwelling older adults ranging from 0.9 to 1.3 m/s [34].

To detect *full-signal* stops, initially, the first GPS record in the trajectory is added to the cluster. Then, each time a GPS record (*P_i_*) is read, three measures are evaluated: (1) the time interval between the last GPS record (*P_i–1_*) in the cluster and *P_i_*, (2) the distance between *P_i–1_* and *P_i_*, and (3) the speed at *P_i_*. If all values are less than their corresponding thresholds (*δ_t_, δ_d_,* and *δ_v_* ), the GPS record (*P_i_*) is added to the cluster. Otherwise, we add the cluster to the list of stops (*S*), set the previous cluster to be the current cluster, and empty the current cluster.

To detect *no-signal* stops, we compute the distance and time interval between each GPS record and its previous record in the trajectory; if the extracted distance is less than the distance threshold and the time interval is more than the time threshold, a cluster of the two GPS records is added to the stop list.

Finally, when a cluster is added to the stop list, the distance and time interval between the centroid of the cluster and the previous cluster is computed; if the values are less than the corresponding thresholds, the two clusters are merged, the previous cluster is set to the new merged cluster, and the current cluster is emptied.

#### Evaluation

**Figure 2:**
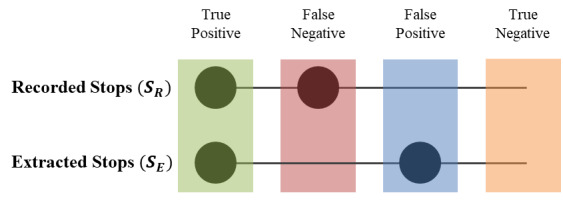
Comparison of the stops recorded in the travel diary versus the stops extracted from the GPS data.

To evaluate the performance of the stop-detection algorithm, the stop points recorded in the travel diary (*S_R_*) are compared with the stop points extracted from the GPS data (*S_E_*). Figure 2 demonstrates different stop conditions. A *true positive* (TP) refers to a stop location recorded in the travel diary that correctly matches with a stop location extracted from the GPS data. We find a match if the distance between the recorded stop and the extracted stop is smaller than 150 meters. A *false negative* (FN) stands for a stop point recorded in the travel diary that is not extracted from the GPS data (ie, the algorithm considers it to be part of a trip or the user forgot to take the GPS device). A *false positive* (FP) refers to a stop point extracted from the GPS data but not recorded in the travel diary. Finally, a *true negative* (TN) occurs when no stop is extracted or recorded.

Using the four stop conditions (ie, TP, TN, FP, and FN), the F1 score is determined to evaluate the performance of the stop-detection algorithm:

*F1 score* = (2 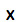
* precision*
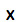
* recall*) / (*precision* + *recall*)

where precision is TP/(TP + FP) and recall is TP/(TP + FN).

### Activity Inference

#### Algorithm

##### Home

**Figure 3:**
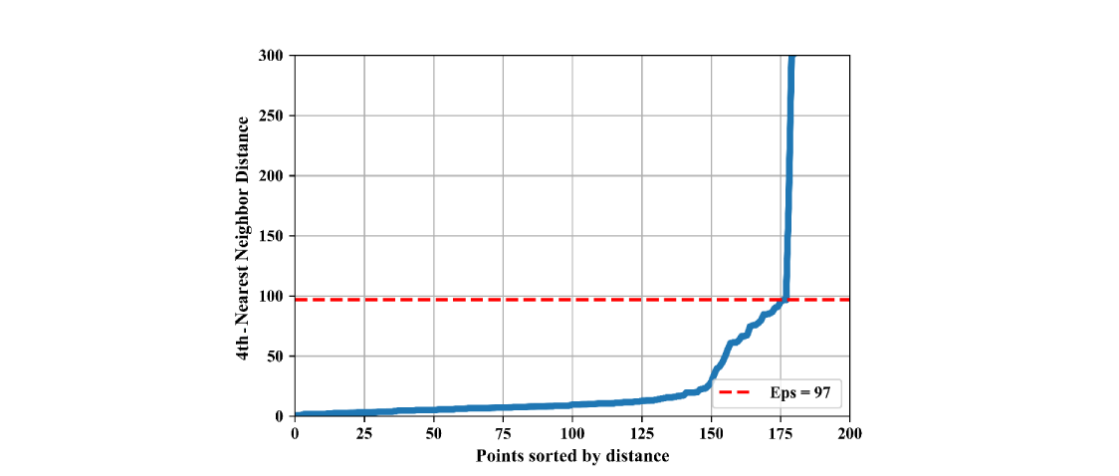
Participant 1's fourth-nearest neighbor distance. *Eps*: cluster radius.

To infer the home location of each participant, the algorithm for density-based spatial clustering of applications with noise (DBSCAN) was used [35]. DBSCAN has been successfully used to find stop points with the most visits in GPS trajectories [36]. Two parameters that affect the results in DBSCAN are the cluster radius (*Eps*) and the minimum number of points required to form a cluster (MinPts). The MinPts parameter was set to 4 as suggested in Ester et al [35] for 2D data. To obtain the optimal *Eps* value for each participant, we draw a k-distance plot for *k*=4 and find the *knee*, which corresponds to a sharp change of gradient along the curve [37]. The fourth-nearest neighbor distance plot for participant 1 is presented in Figure 3. The knee point, which represents a change in density among the stop points, is selected as the optimal *Eps*. Table 1 demonstrates the optimal *Eps* value for each participant.

**Table 1:** Optimal cluster radius (*Eps*) value for each participant.

Participant	*Eps*, m
1	97
2	94
3	59
4	32
5	95

##### Other Activities

The activity-inference algorithm takes as input the list of stops and outputs the most probable activity type at each stop location. For each stop point (*S_i_*), a Nearby Search was invoked from the Google Places application programming interface (API). The search returned a list of places within a 150-meter radius of the *S_i_*. Then, for each place, a Place Details search was invoked, which returned details including place name, type, and opening hours. The place was only considered if it was open during the stop. Next, the place types were mapped into activity types according to Table 2. If the Nearby Search returns nothing or if the place type does not fall into any of the listed categories, it would be placed into the *Other* category. To find the most probable activity type at each stop, the gravity model was implemented [27]. This model associated a probability to each possible activity by taking into account the distance of each place from the stop and the general characteristics of the stop location. For example, if a stop is in an area with many places that are mapped to food and few places that are mapped to medical services, the gravity model gives more weight to food as compared to medical services.

**Table 2:** Mapping between place types and activities.

Activity category	Place type
Food	Bakery, bar, cafe, food, meal takeaway, restaurant, and meal delivery
Daily shopping	Grocery and supermarket
Shopping	Bookstore, clothing store, convenience store, hardware store, electronics store, furniture store, shopping mall, liquor store, pet store, shoe store, and department store
Services	ATM (automatic teller machine), bank, car rental, car repair, finance, insurance, gas station, travel agency, post office, accounting, beauty salon, courthouse, and laundry
Leisure	Bowling alley, casino, library, movie rental, movie theater, museum, park, spa, stadium, and lodging
Medical services	Dentist, hospital, pharmacy, physiotherapist, chiropractor, psychologist, naturopath, walk-in clinic, sleep lab, LifeLabs^a^, and Dynacare^a^
Religious	Church, Hindu temple, synagogue, and mosque
Sport	Gym and YMCA

^a^LifeLabs and Dynacare are medical laboratory services companies based in Ontario.

#### Evaluation

From the TP stops, the ones annotated by the activity-inference algorithm are compared to the ones declared by the participant in the travel diary. For the activities annotated as *Activity at Home*, the distances between the participant’s actual home location and extracted home locations are computed.

## Results

### Participants

A total of 5 cognitively intact, community-dwelling individuals completed 4 weeks (ie, 28 days) of GPS monitoring and travel diary recording. Table 3 presents the summary statistics of the travel diary recordings for each participant.

The average age of participants in the study sample was 73 years (SD 6) (range 68-80); participants lived within the Greater Toronto Area and were all active drivers. Study participants had an average Montreal Cognitive Assessment (MoCA) score of 27.8 (SD 1.8). All 5 participants received a score of 6 in ADL and 8 in IADL, indicating the highest level of function. For more details of the sample’s demographic characteristics, including the Walk Score [38], refer to Table 4.

The sample of 5 consisted of 4 retired older adults (80%) (Participants 2-5) and 1 older adult (20%) with a part-time job (Participant 1). It should be noted that although employed, Participant 1 was not working during the 4 weeks of the study.

**Table 3:** Summary statistics of 4 weeks of travel diary recordings for the participants.

Participant	Number of stops	Stops per day, mean (SD)
1	117	5.6 (2.5)
2	89	3.4 (1.5)
3	88	3.5 (1.8)
4	124	4.6 (1.9)
5	108	4.2 (1.9)

**Table 4:** Sample demographic characteristics.

Participant	Age in years	MoCA^a^ score	Sex	ADL^b^ score	IADL^c^ score	Employment status	Driving status	Walk Score^d^
1	68	26	Female	6	8	Employed part time	Driving	65
2	70	26	Female	6	8	Retired	Driving	30
3	78	30	Female	6	8	Retired	Driving	57
4	68	29	Male	6	8	Retired	Driving	94
5	80	28	Female	6	8	Retired	Driving	69

^a^MoCA: Montreal Cognitive Assessment.

^b^ADL: activities of daily living. ADL scores ranged from 0 (lowest level of function) to 6 (highest level of function).

^c^IADL: instrumental activities of daily living. IADL scores ranged from 0 (lowest level of function) to 8 (highest level of function).

^d^Walk Score is a measure of access to walkable amenities, ranging from 0 (Car-Dependent) to 100 (Walker's Paradise) [38].

### Stop Detection

**Figure 4:**
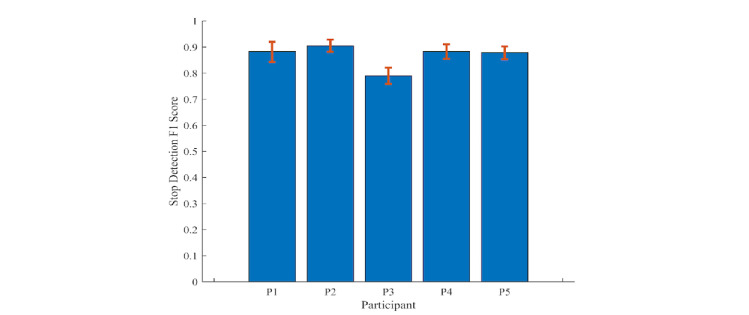
Stop-detection F1 scores for participants 1 to 5 (P1-P5).

We reached a global stop-detection F1 score of 87% for all participants. The F1 score of each participant was computed and presented in Figure 4. The scores suggest that destinations of individuals can be detected with reasonable accuracy.

**Figure 5:**
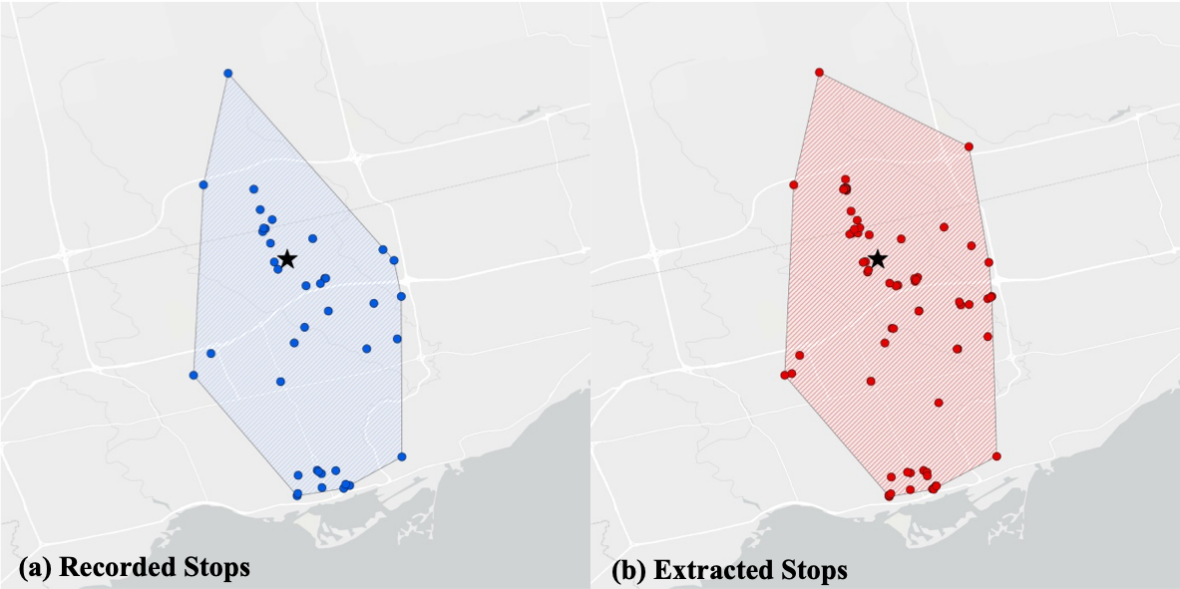
Comparison of (a) stops recorded in the travel diary versus (b) stops extracted from the GPS data for Participant 1.

We further evaluated the stop detection by analyzing the mobility patterns. Figure 5 shows the distribution of stop locations recorded in the travel diary (left) versus the ones extracted from GPS data (right) for Participant 1 over the 4 weeks of the study. Additionally, an envelope was built around all stop points using the convex hull algorithm to represent the extent of travel into the environment (ie, life-space area). From Figure 5, it is clear that the two distributions are similar, although some stop points are missing in the diary plot.

### Home and Activity Inference

Table 5 presents the distances between the participants’ actual home locations and extracted home locations. On average, the extracted home locations were within a 40.18-meter (SD 1.18) distance of the actual home locations.

For all activity types including activities at *home*, F1 scores were obtained, which determine the percentage of activities correctly classified with respect to the activities recorded in the travel diary. Each participant’s score is presented in Table 6. A global F1 score of 86% was reached for all participants.

**Table 5:** Distances between the actual home locations and extracted home locations.

Participant	Distance, m
1	6.60
2	50.3
3	19.9
4	20.4
5	76.7

**Table 6:** Activity inference’s F1 score.

Participant	F1 score	Number of stops
1	0.89	74
2	0.90	79
3	0.86	101
4	0.79	95
5	0.86	87

**Figure 6:**
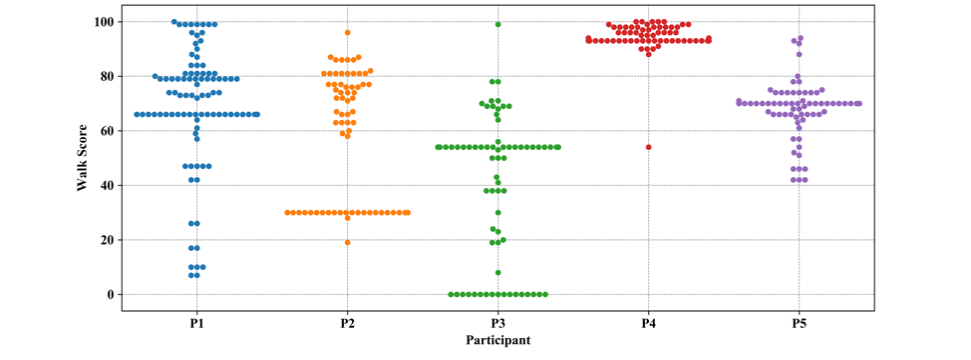
Distribution of Walk Scores of each participant’s (P) destinations, as calculated by Walk Score [38]. A score of 0-24 or 25-49 is considered Car-Dependent, a score of 50-69 is considered Somewhat Walkable, a score of 70-89 is considered Very Walkable, and a score of 90-100 is considered Walker's Paradise.

The scores suggest that we can infer activity types with reasonable accuracy for all participants. It can be demonstrated that the algorithm reached its lowest score for Participant 4. To understand the reason behind this, the neighborhood characteristics of each participant’s destinations were examined using Walk Score, a measure of access to walkable amenities, ranging from 0 (Car-Dependent) to 100 (Walker's Paradise) [38]; the distribution of scores are shown in Figure 6. It can be demonstrated that for Participant 4, all destinations are located in neighborhoods with Walk Score values of greater than 80.

**Figure 7:**
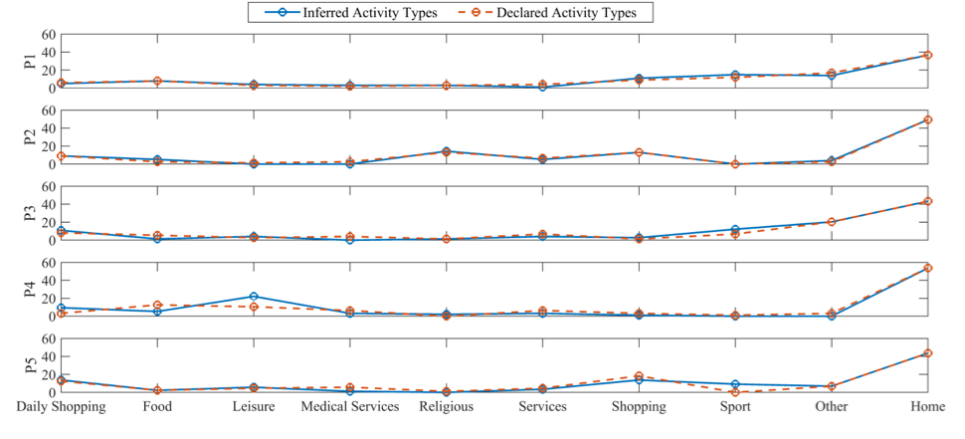
Comparison of the number of activity types in the travel diary versus the ones inferred from the GPS data for Participants 1 to 5 (P1-P5).

Furthermore, for personalized evaluation of the activity-inference algorithm, plots showing the percentage of activities that were inferred from the GPS data and declared in the travel diary for all 5 participants and each activity category are presented in Figure 7. In general, the percentage of inferred activities and percentage of declared activities follow a similar trend.

In order to further demonstrate the effects of neighborhood characteristics on the performance of activity inference, the out-of-home destinations were first placed into five groups according to their Walk Score [39]: (1) Walker’s Paradise (90-100), where most errands can be accomplished on foot, and many people get by without owning a car; (2) Very Walkable (70-89) areas, where it is possible to accomplish most errands without driving; (3) Somewhat Walkable (50-69) areas, where some amenities are within walking distance, but many daily errands still rely on public transportation or driving; (4) Car-Dependent (25-49) areas, where a few destinations are within walking distance, but most activities require driving or public transportation; and (5) Very Car-Dependent (0-24), where no neighborhood destinations are within walking range. Then, the average F1 score of the activity-inference algorithm in each group was determined. Table 7 illustrates that by moving from neighborhoods with a high density of amenities to neighborhoods with a low density of amenities, the performance of the activity-inference algorithm improves.

**Table 7:** Effects of Walk Score on performance of the activity-inference algorithm.

Category	Walk Score^a^	F1 score	Number of stops
Walker’s Paradise	90-100	0.58	58
Very Walkable	70-89	0.76	87
Somewhat Walkable	50-69	0.83	46
Car-Dependent	25-49	0.79	22
Very Car-Dependent	0-24	0.96	24

^a^Walk Score is a measure of access to walkable amenities [38].

## Discussion

### Principal Findings

This paper presents a framework that allows classification of semantic aspects of outdoor mobility including destination and activity type. This framework is complementary to the GPS-based mobility indicators used in the literature to assess spatial and temporal facets of outdoor mobility and can enhance the understanding of older adults’ outdoor mobility behavior.

The results show that, on average, 86% of activity types, including home activities, recorded in the travel diaries were correctly inferred. The performance of the activity-inference algorithm, however, depends on the neighborhood characteristics of destinations. If a destination is in a neighborhood with a high Walk Score (ie, Very Walkable or Walker’s Paradise) where places are densely positioned, the algorithm can have difficulty identifying the correct places and the corresponding activity types; however, for destinations in less-walkable neighborhoods with a low Walk Score (ie, Very Car-Dependent, Car-Dependent, or Somewhat Walkable), the algorithm performed better. This pattern further hinders the performance of the algorithm when a participant’s primary residence is in a high–Walk Score neighborhood, where their destinations are mostly concentrated within the same area, as illustrated with Participant 4’s results in Figure 6. This renders the algorithm unable to precisely draw inferences, given the high-density life space, ultimately resulting in a lower F1 score. Furthermore, looking into the Walk Score of each participant’s destinations (see Figure 6), it can be observed that among our 5 participants, Participant 4’s destinations were positioned in locations with a higher Walk Score, and the algorithm reached the lowest activity-inference F1 score for this participant.

It is important to note that there are challenges associated with using travel diaries as ground truth. Participants’ compliance with the diaries tends to decline over time. Due to the burden of manual data recording, participants may avoid or forget to record trips that are too short or that are made at inconvenient times. By examining the distributions of recorded and extracted stop locations in Figure 5, it can be observed that more stops were extracted from the GPS data in comparison to the stops recorded in the diary, suggesting that some stops in the GPS data were not recorded in the travel diary.

Furthermore, travel diaries included the street addresses of the destinations. In order to build ground truth of the destinations, we first geocoded each street address using the Google Maps API. Given a street address, Google Maps API returned the latitude and longitude coordinates. However, this process is not always straightforward. For example, in the cases where the recorded address in the diary is incomplete, the correct coordinate may not be determined. To minimize these ambiguities, during the digitizing process of the travel diaries, the recorded addresses were verified. Since a verified address corresponds to a unique geographical coordinate, the destinations that were visited multiple times had the exact same latitude and longitude coordinates and are completely overlapping in Figure 5. This is not the case for the stops extracted from the GPS data. The GPS device does not record exactly the same coordinate for a unique destination. Even if the participant is staying at the same location for some time, the GPS records can vary by up to 15 meters. Therefore, in the GPS data, the coordinates corresponding to the same physical location can vary. This can be clearly observed in Figure 5 where there are multiple stops in close proximity belonging to the same location.

The mapping between the place types and activity categories is another important issue. The activity categories are inferred based on the place types available on the Google Maps. However, there are some occasions where the activities conducted at a specific place cannot be uniquely determined. For example, playing golf with some friends can be considered as *Sport* or *Leisure* activity. On the other hand, due to the limitations of GPSs in indoor settings, for some multipurpose locations the correct activity type cannot be inferred. For example, a movie theater inside a shopping center can be identified as a *Shopping* activity instead of a *Leisure* activity. This issue can be addressed in future studies by having a hybrid system that relies on a Wi-Fi positioning system for indoor tracking and GPS for outdoor tracking.

Our proposed model outperforms the existing activity-inference techniques. One major advantage of the proposed model is that it does not require any prior information regarding users' most-visited destinations. Most of the available techniques for inferring activity types from raw trajectories involve some initial data collection at the time of recruitment. These studies collect the addresses of each participant’s most-visited locations, such as home, workplace, school, or the frequently used grocery stores [20-22], and claim that these locations yield more than 60% of each participant’s activities [21]. Our presented model, however, is fully autonomous and uses a density-based clustering method to extract the most-visited destinations of each participant. This improvement is particularly important for exploring activity profiles of the older adult population because their most-visited destinations are not always an educational establishment or workplace. Another advantage is that while most of the prevailing techniques use a small list of points of interest to infer activity types [20,22], our model takes advantage of the Google Places API system, which is currently the most comprehensive dataset of points of interest. Additionally, while most studies use a uniform probability measure to select the most appropriate activity category [21,22,25], our model implemented a gravity-based approach similar to the method introduced in Furletti et al [27] to select the most probable activity category for each destination.

Furthermore, most studies that present empirical results of activity-inference methods only focus on car trips [24,26,27]. The GPS data used in this study were collected by participants during their out-of-home trips using various modes of transportation. It is worth noting that although the gravity-based probability measure used to infer activity types in our study was similar to the approach introduced in Furletti et al [27], our results achieved a higher accuracy. This is because in Furletti et al [27], they used positioning data collected by tracking devices installed in cars. In this case, the identification of the stop locations can become problematic because a car usually cannot enter inside the stop location, meaning that the participant needs to park the car and then walk to the destination, which will not be tracked by the GPS device. In our study, however, the GPS devices were being carried by the participants inside all the stop locations they visited. Furthermore, our activity-inference algorithm also discusses the detection of the home location, which was not included in Furletti et al [27].

### Limitations

The findings should be understood in light of some inherent limitations within this study, which can be addressed in future research. First, the small sample size in this study, although effective for demonstrating the feasibility of the developed algorithms [31], is not representative of the population and is, thus, not suitable for statistical analysis of the population. Further studies on larger sample sizes are required in order to comprehensively analyze the activity profiles of older adult populations. Second, a cutoff of 65 years of age was used in this study, resulting in a sample age range of 68-80 years. This wide age range along with the small sample size prohibited analysis on age-related changes in the activity profiles of older adults. Future studies can divide the older adult population into three subgroups—the young-old (65-74 years), the middle-old (75-84 years), and the old-old (over 85 years)—and investigate the mobility and activity profile of each subgroup separately. Third, all the participants were from the Greater Toronto Area. Further research is required on older adults from a set of representative locations that reflect the climatic, socioeconomic, and geographic diversity of the older adult population. Finally, due to recruitment challenges, our sample contained an unbalanced sex distribution (ie, 1 male and 4 females), which prohibited any statistical analysis by sex or gender. Future studies should examine gender differences, since they may be attributable to a variety of interrelated factors, including differences in perceptions of safety and cultural norms regarding outdoor mobility.

### Conclusions

In environmental gerontology research, GPS devices are becoming increasingly more common to accurately and continuously measure older adults’ outdoor mobility, thereby addressing limitations of traditional self-reported measures, such as recall biases. Outdoor mobility, however, is a multidimensional concept and it is challenging to characterize it comprehensively with only spatiotemporal indicators derived from GPS data.

In this paper, we extend the literature on older adults’ mobility models through development and validation of a framework that relies on GPS data to capture older adults’ travel destinations (ie, stop points) and activity types. We have performed a comparison with ground truth based on travel diaries, and we have evaluated in detail the performance of the implemented stop-detection and activity-inference algorithms. Our results indicate that it is possible to extract destinations and infer activity types from GPS data with reasonable accuracy.

This paper encourages incorporation of GPS-based mobility indicators that reflect the semantic dimension of individuals’ outdoor mobility into future health- and aging-related research. This approach fosters a better understanding of what aspects of mobility are key to healthy aging. It also shows great potential in examining the impact of interventions and long-term monitoring of social connection, functionality, and quality of life. Future research should aim to utilize GPS technology to assess older adults’ transportation modes in order to provide insights about different ways of conceptualizing older adults’ environmental exposure.

## Abbreviations

ADL: activities of daily living
AGE-WELL NCE: Aging Gracefully across Environments using Technology to Support Wellness, Engagement and Long Life Networks of Centres of Excellence
API: application programming interface
ATM: automatic teller machine
CCNA: Canadian Consortium on Neurodegeneration in Aging
DBSCAN: density-based spatial clustering of applications with noise
EMA: ecological momentary assessment
*Eps*: cluster radius
FN: false negative
FP: false positive
IADL: instrumental activities of daily living
MinPts: minimum number of points required to form a cluster
MoCA: Montreal Cognitive Assessment
TN: true negative
TP: true positive
